# Supplementary material for: Directed differentiation of mouse pluripotent stem cells into functional lung-specific mesenchyme
Source: Nat Commun. 2023 Jun 13;14:3488. doi: 10.1038/s41467-023-39099-9 (PMC10264380; doi:10.1038/s41467-023-39099-9)
Supplement: Supplementary file 1 — Supplementary Information [file 41467_2023_39099_MOESM1_ESM.pdf]

Directed differentiation of mouse pluripotent stem cells into functional lung-specific mesenchyme

Andrea B. Alber[1,2], Hector A. Marquez[1,2], Liang Ma[1,2], George Kwong[1,2], Bibek R. Thapa[1,2], Carlos Villacorta-Martin[1], Jonathan Lindstrom-Vautrin[1], Pushpinder Bawa[1], Feiya Wang[1], Yongfeng Luo[3], Laertis Ikonomou[4,5], Wei Shi[6], Darrell N. Kotton[1,2*]

[1]Center for Regenerative Medicine of Boston University and Boston Medical Center, Boston, MA 02118, USA
[2]The Pulmonary Center and Department of Medicine, Boston University School of Medicine, Boston, MA 02118, USA
[3]Department of Surgery, Children's Hospital Los Angeles, Keck School of Medicine, University of Southern California, Los Angeles, CA 90027, USA
[4]Department of Oral Biology, School of Dental Medicine, University at Buffalo, Buffalo, NY 14260, USA
[5]Division of Pulmonary, Critical Care and Sleep Medicine, Department of Medicine, Jacobs School of Medicine and Biomedical Sciences, University at Buffalo, Buffalo, NY 14215, USA
[6]Division of Pulmonary, Critical Care, and Sleep Medicine, Department of Internal Medicine, University of Cincinnati College of Medicine, Cincinnati, OH 45267, USA
*Corresponding author (dkotton@bu.edu)

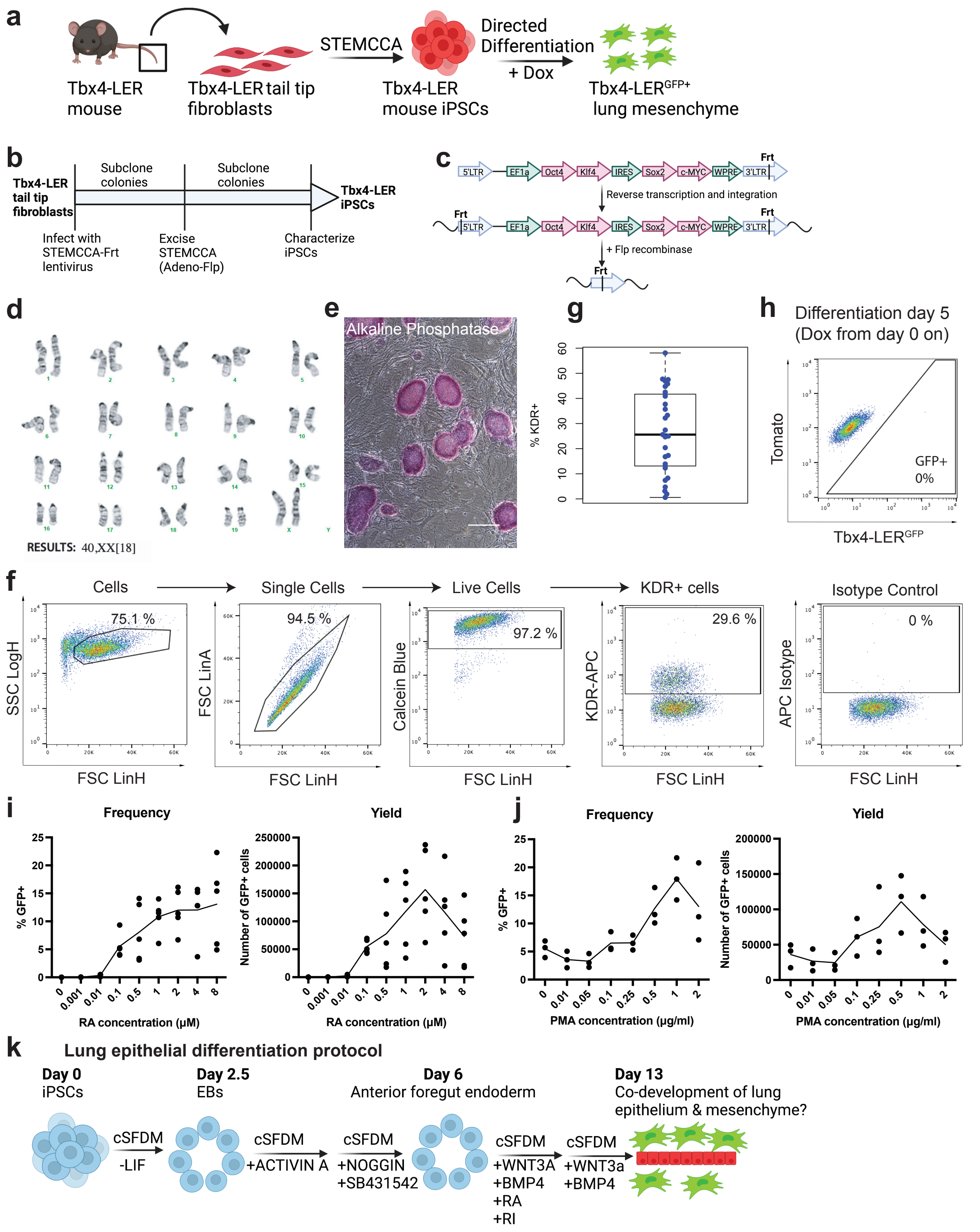

Supplementary Figure 1: Reprograming of Tbx4-LER^{GFP} tail tip fibroblasts and directed lung mesenchymal differentiation.

a: Overview of reprogramming procedure and generation of induced lung mesenchyme. Created with BioRender.com. **b:** Schematic showing the generation of iPSCs from tail tip fibroblasts. Created with BioRender.com. **c:** Schematic showing STEMCCA lentiviral cassette before and after excision of the Frt-flanked STEMCCA sequence. Created with BioRender.com. **d:** Karyotyping results showing normal karyotype of Tbx4-LER iPSC line. **e:** Alkaline phosphatase stain of Tbx4-LER iPSC line. Scale bar = 100 µm. **f:** Example of gating strategy and representative flow cytometry plot showing KDR stain and isotype on day 5 of differentiation. **g:** Percentage of KDR+ cells on day 5 of differentiation (lateral plate mesoderm stage). Each dot represents an individual differentiation experiment, box plots indicate median (middle line), 25th and 75th percentile (box), and 5th and 95th percentile (whiskers). N=27. **h:** Flow cytometry plot showing expression of GFP and Tomato on day 5 of differentiation, when dox was kept in the medium from day 0 on. **i:** Frequency and yield of GFP+ cells using various RA concentrations from 0 to 8 µM. **j:** Frequency and yield of GFP+ cells using various PMA concentrations from 0 to 2 µg/ml. **k:** Protocol for the directed differentiation of iPSCs towards the lung epithelial lineage. cSFDM = complete serum-free differentiation medium, RA = retinoic acid, RI = ROCK inhibitor (Y-27632). Created with BioRender.com.

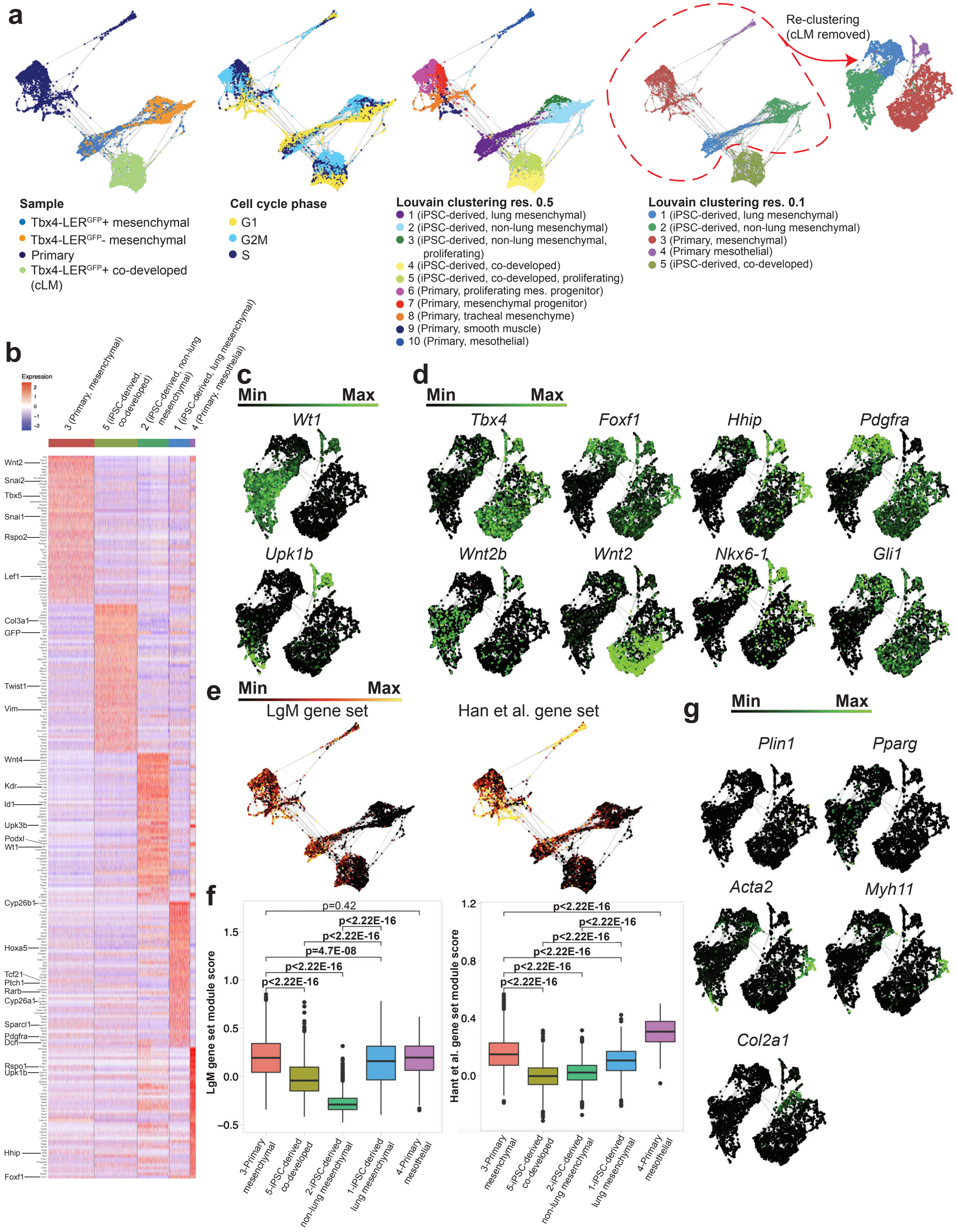

Supplementary Figure 2: scRNA-seq of iPSC-derived and primary lung mesenchyme.

a: SPRING plot overview, cell cycle phase and Louvain clustering (resolution 0.5 and 0.1) of the full scRNA-seq dataset including primary E12.5 cells, Tbx4-LER^{GFP}+/- generated with the lung mesenchymal directed differentiation protocol ("mesenchymal"), as well as Tbx4-LER^{GFP}+ cells generated by co-development ("co-developed"). Cluster 5 (co-developed lung mesenchyme, cLM) was removed for plots shown in panels c, d and g, as well as main Fig. 2. **b:** Heatmap showing top 50 enriched genes in clusters 1-5 (based on Louvain clustering resolution 0.1). Genes of interest are highlighted in larger font. **c:** SPRING plots showing expression of mesothelial markers *Wt1* and *Upk1b* in iPSC-derived and primary cell clusters. **d:** SPRING plots showing expression of lung mesenchymal markers of interest in iPSC-derived and primary cell clusters. **e:** SPRING plots showing expression of the LgM and Han et al. gene sets in primary E12.5 cells, Tbx4-LER^{GFP}+/- generated with the directed lung mesenchymal differentiation protocol ("mesenchymal"), as well as Tbx4-LER^{GFP}+ cells generated by co-development (cLM). **f:** Quantification of the expression of the LgM and Han et al. gene sets in clusters 1-5. Box plots indicate median (middle line), 25th and 75th percentile (box), 5th and 95th percentile (lines), as well as outliers. P values were determined by unpaired, two-tailed Student's t test. Significant ($p<0.05$) p values are highlighted in bold font. N = 1985 (3 – Primary Mesenchymal), 1847 (5 – iPSC-derived co-developed), 1372 (2 – iPSC-derived, non-lung mesenchymal), 882 (1 – iPSC-derived lung mesenchymal), 223 (4 – Primary mesothelial). **g:** SPRING plot showing expression of mature mesenchymal lineage markers in iPSC-derived and primary cell clusters (*Plin*, *Pparg* = adipogenic/lipofibroblast; *Col2a1* = chondrogenic; *Acta2*, *Myh11* = smooth muscle).

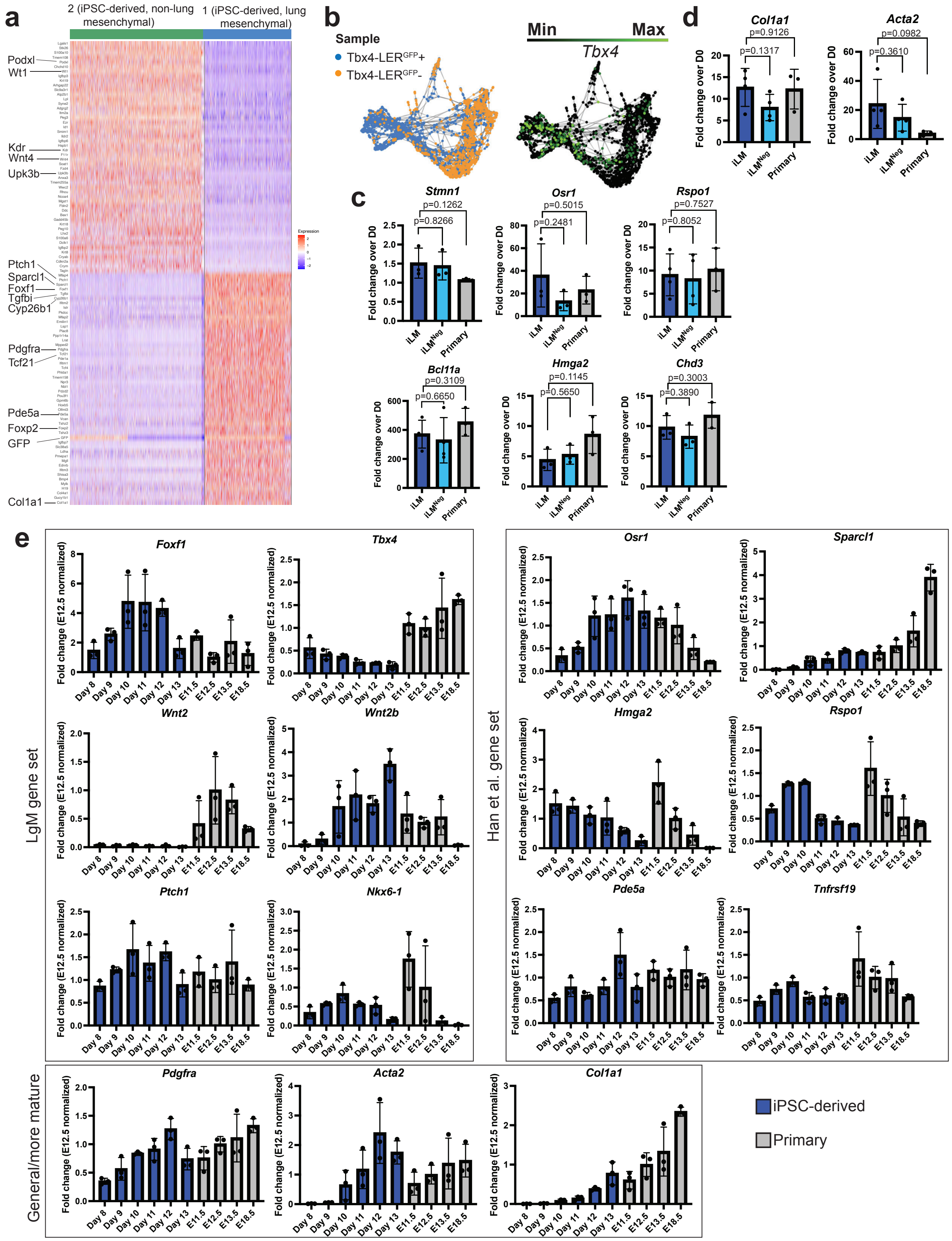

Supplementary Figure 3: Transcriptomic analysis of iPSC-derived and primary lung mesenchymal cells.

a: Heatmap showing top 50 enriched genes in pairwise comparison between iPSC-derived clusters 1 and 2. Genes of interest are highlighted in larger font. **b:** SPRING plot showing expression of *Tbx4* transcripts in iPSC-derived Tbx4-LER^{GFP}+ and – cells generated by directed lung mesenchymal differentiation. **c:** RT-qPCR showing fold change expression relative to day 0 iPSCs of lung mesenchyme markers from Han et al.'s gene set in induced lung mesenchyme (iLM) and iLM^{Neg} cells compared to primary lung mesenchyme from E12.5 embryos. Tbx4-LER^{GFP}+/- cells were collected on differentiation day 13 and dox was added from day 5 on. N = 3. **d:** RT-qPCR showing fold change expression relative to day 0 iPSCs of *Col1a2* and *Acta2* in induced lung mesenchyme (iLM) and iLM^{Neg} cells compared to primary lung mesenchyme from E12.5 embryos. Tbx4-LER^{GFP}+/- cells were collected on differentiation day 13 and dox was added from day 5 on. N = 3 for "Primary", N = 4 for "iLM" and "iLM^{Neg}". **e:** RT-qPCR showing fold change expression relative to day 0 iPSCs of lung mesenchyme markers in iPSC-derived Tbx4-LER^{GFP}+ every day from day 8-13 of differentiation, as well as in primary embryonic lung mesenchyme from various time-points. For iPSC-derived cells dox was added 2 days prior to collection. N = 3.
All bars show mean ± sd. P values were determined by unpaired, two-tailed Student's t test. Significant (p<0.05) p values are highlighted in bold font.

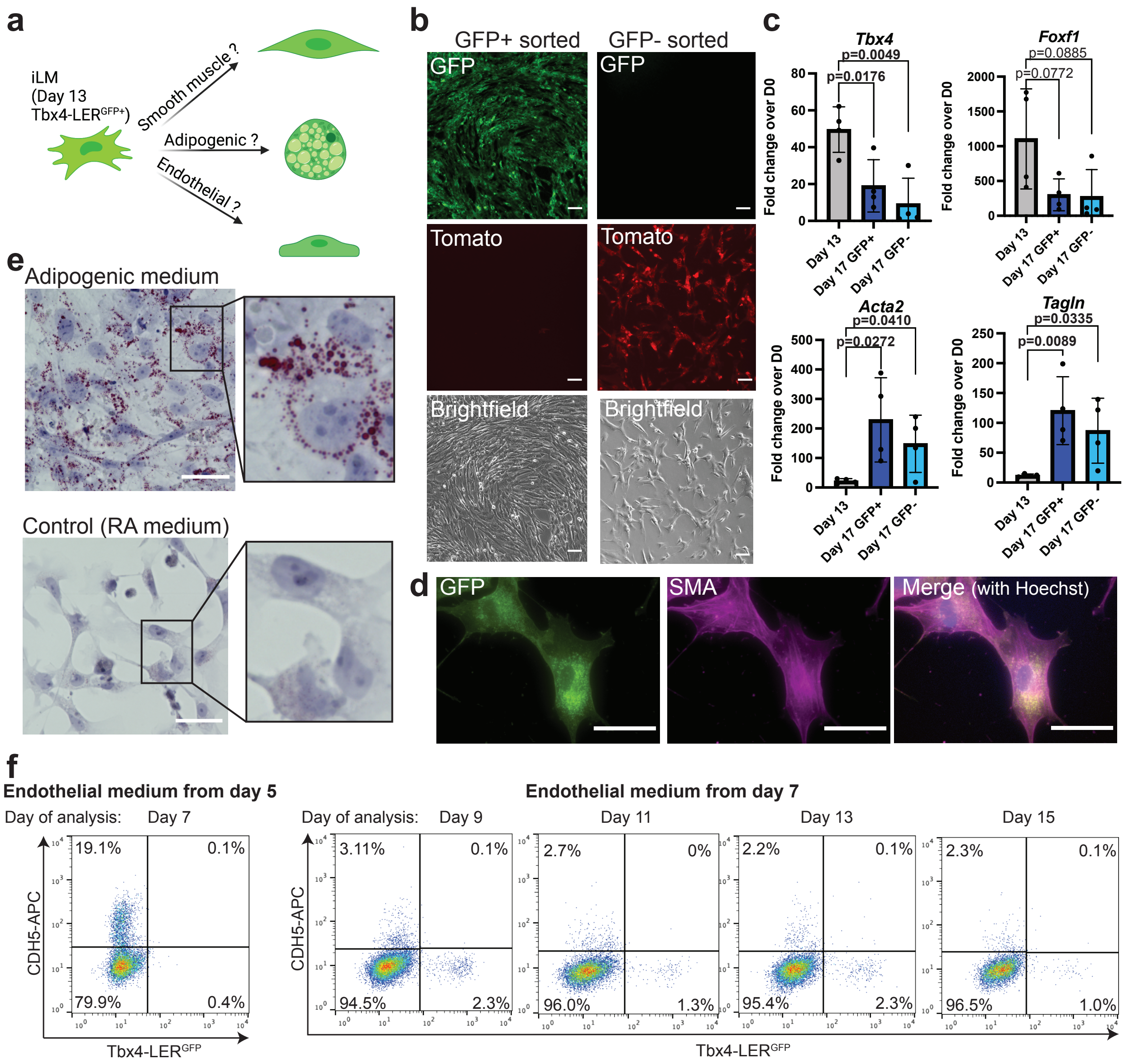

Supplementary Figure 4: Differentiation of induced lung mesenchymal progenitors towards more mature general mesenchymal lineages.

a: Schematic of mesenchymal lineage differentiations performed. Created with BioRender.com. **b:** Representative image showing morphology of Tbx4-LER^{GFP}+ vs Tbs4-LER^{GFP}- cells sorted on day 13 of differentiation and subsequently cultured in smooth muscle differentiation medium for 4 days. Scale bars = 100 µm. **c:** RT-qPCR showing fold change expression relative to day 0 iPSCs of early lung mesenchyme markers *Tbx4* and *Foxf1*, as well as smooth muscle markers *Acta2* and *Tagln* on day 13 of differentiation (D13 = day 13 unsorted cells), and after 4 days of culture in smooth muscle differentiation medium (D17 GFP+/- = Tbx4-LER^{GFP}+/- cells sorted on day 13 and subsequently cultured in smooth muscle medium for 4 days). N = 4, Bars show mean ± sd. P values were determined by unpaired, two-tailed Student’s t test. Significant ($p<0.05$) p values are highlighted in bold font. **d:** Immunofluorescence image showing expression of GFP and smooth muscle actin (SMA) in Tbx4-LER^{GFP}+ cells that were sorted on day

13 and cultured in smooth muscle medium for 4 days. Scale bars = 100 µm. **e:** Oilred O and hematoxylin stain of cells sorted for Tbx4-LER^{GFP}+ on day 13 and cultured in adipogenic medium for 9 days. Control cells were kept in lung mesenchyme medium (cSFDM+RA). Scale bars = 100 µm. **f:** Flow cytometry analysis showing CDH5 cell surface expression after culture in endothelial medium (cSFDM+VEGF). Left panel: endothelial medium added on day 5 of differentiation, cells analyzed on day 7. Right panel: endothelial medium added on day 7 of differentiation and cells analyzed on days 9, 11, 13 and 15.

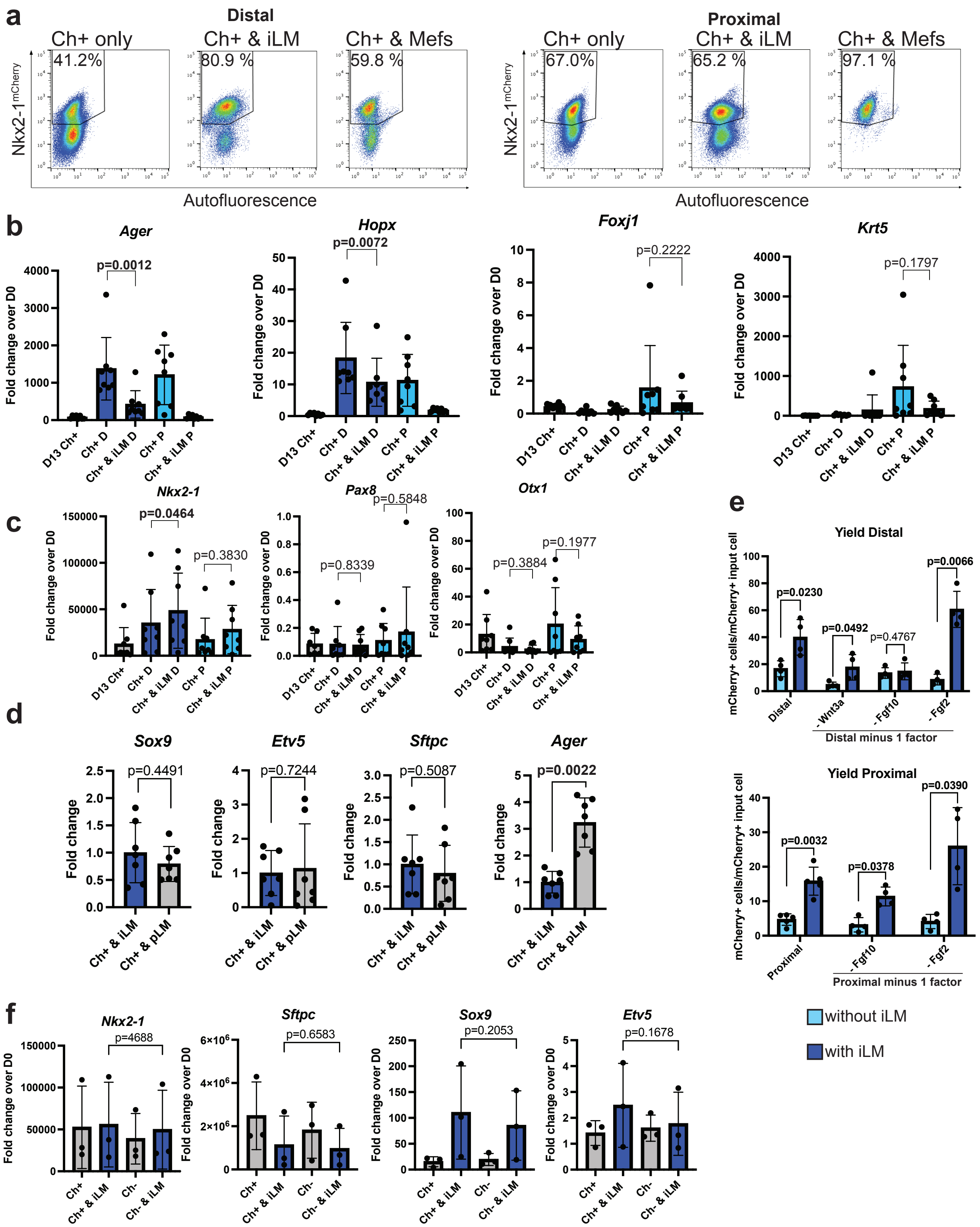

Supplementary Figure 5: Gene expression analysis of co-cultured lung epithelial progenitors.

a: Representative flow cytometry plots showing percentage of Nkx2-1^{mCherry}+ cells for Nkx2-1^{mCherry}+ cells cultured alone, with iLM or with MEFs for 7 days in distal or proximal medium. Cells were pre-gated for single cells, live cells, GFP- and EPCAM+ cells. **b:** RT-qPCR data showing fold change expression relative to day 0 iPSCs of additional distal and proximal lung/airway markers in Nkx2-1^{mCherry}+ cells before (grey) and after co-culture in distal (dark blue) and proximal (light blue) medium. N = 8. **c:** RT-qPCR data showing fold change expression relative to day 0 iPSCs of *Nkx2-1*, *Pax8*, and *Otx1* in Nkx2-1^{mCherry}+ cells before (grey) and after co-culture in distal (dark blue) and proximal (light blue) medium. N = 8. **d:** RT-qPCR data showing fold change expression relative to Ch+&iLM sample of *Sox9*, *Etv5, Sftpc*, and *Ager* in Nkx2-1^{mCherry}+ cells after co-culture with iLM (Ch+&iLM) or primary E12.5 lung mesenchyme (Ch+&pLM). N=7. **e:** Yield (i.e. number of Nkx2-1^{mCherry}+ cells per day 13 Nkx2-1^{mCherry}+ input cell) for Nkx2-1^{Cherry}+ cells cultured without or with iLM in distal or proximal medium, and upon removal of one growth factor per condition. N=4. **f:** Expression relative to day 0 iPSCs of distal lung markers in day 20 Nkx2-1^{mCherry}+ cells after distal culture. Day 13 Nkx2-1^{mCherry}+/- cells (Ch+/Ch-) were sorted on day 13 of differentiation and cultured in distal medium either alone (grey) or with iLM (blue). N = 3.
All bars show mean ± sd. P values were determined by paired, two-tailed Student's t test. Significant ($p<0.05$) p values are highlighted in bold font.

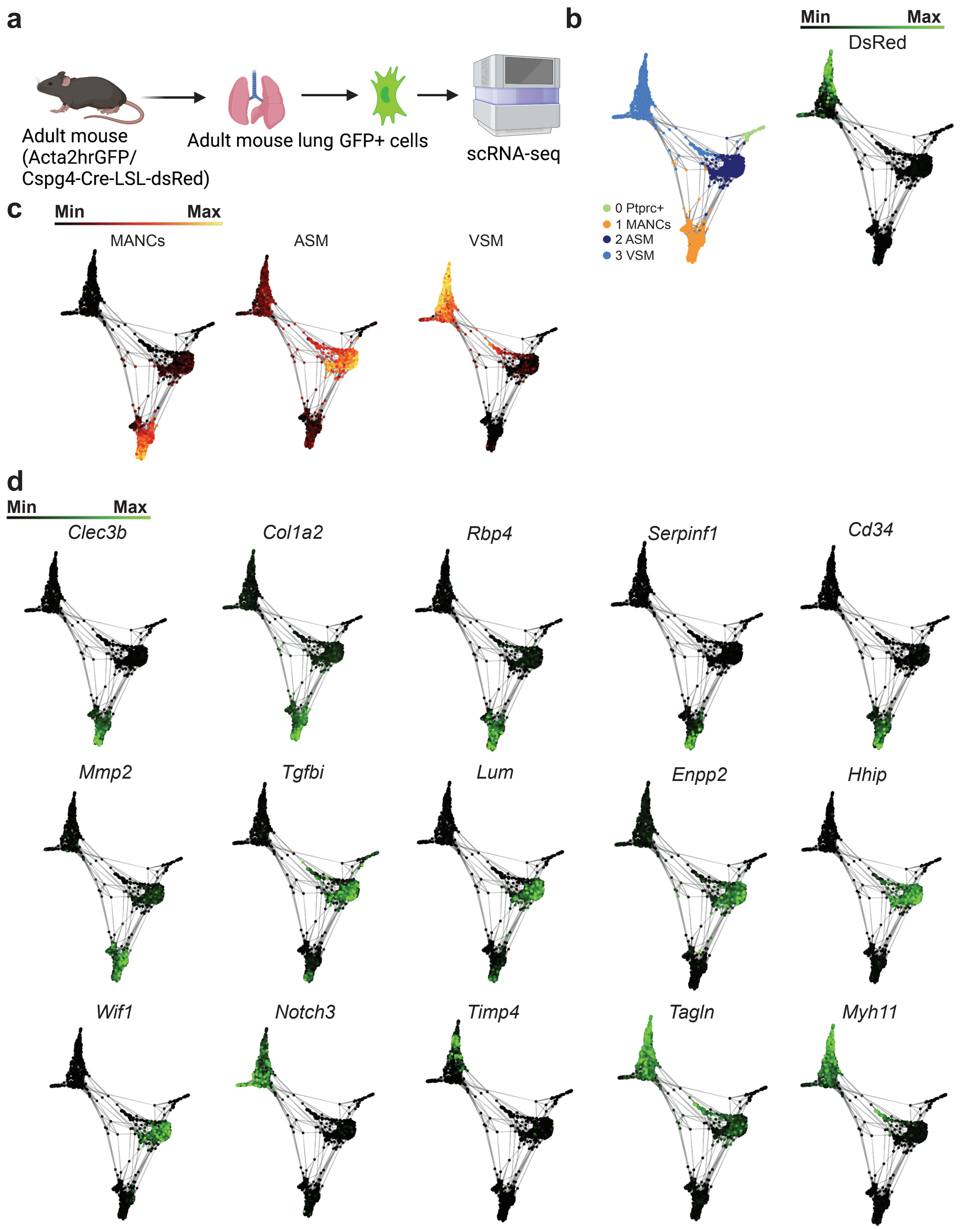

Supplementary Figure 6: scRNA-seq of adult Acta2^{GFP}+ cells.
a: Schematic of scRNA-seq of Acta2^{GFP}+ cells from adult mouse lungs. Created with BioRender.com. **b:** SPRING plots showing Louvain clustering (resolution 0.25) of mouse

lung Acta2^{GFP}+ sorted cells and expression of DsRed in scRNA-seq dataset. **c:** SPRING plots showing overlay of gene sets consisting of top50 expressed genes in mesenchymal alveolar niche cells (MANCs), airway smooth muscle (ASM), and vascular smooth muscle (VSM) cells from Zepp et al., 2021. **d:** SPRING plots showing expression of MANC, ASM and VSM markers used for RT-qPCR in scRNA-seq dataset.

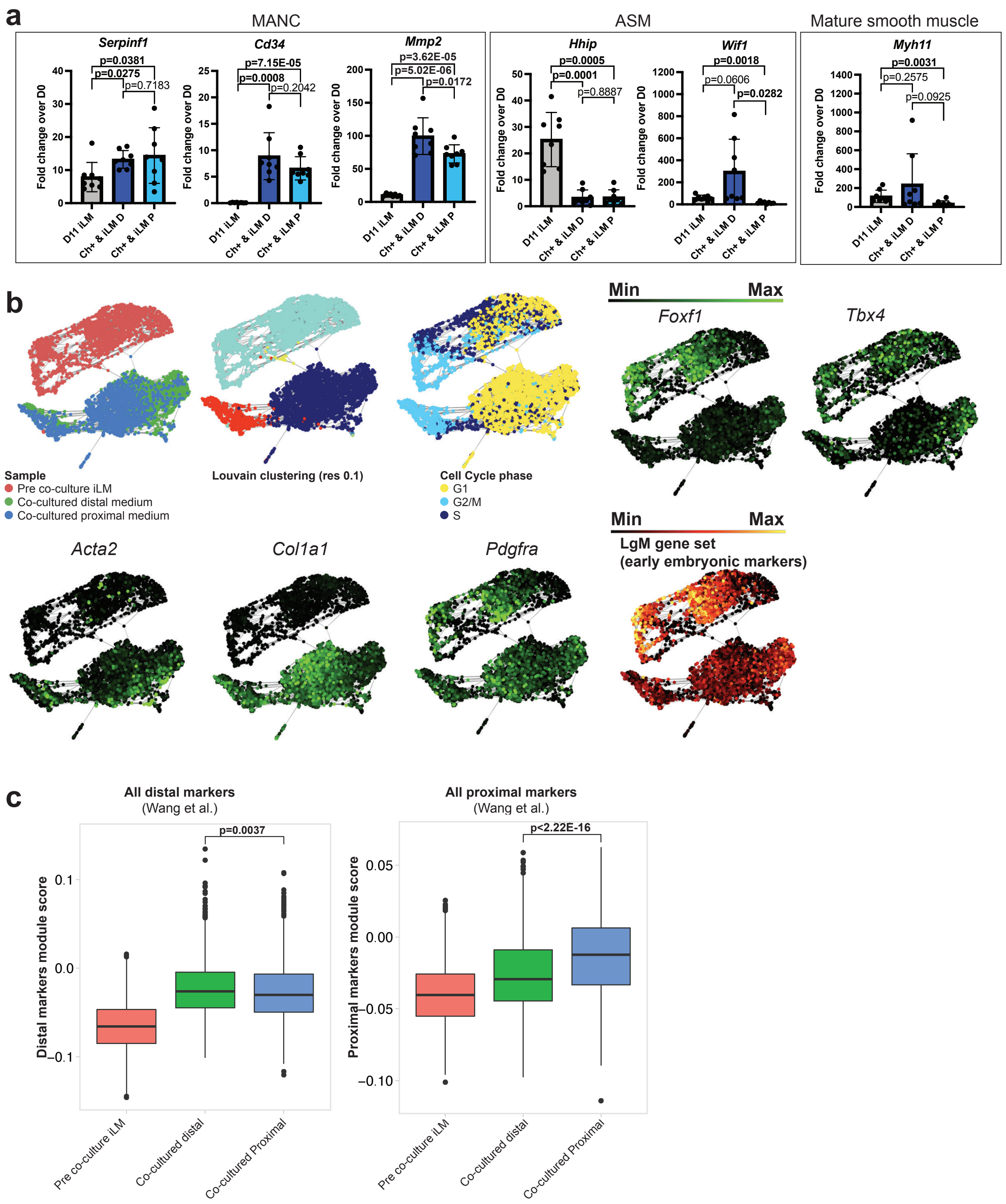

Supplementary Figure 7: scRNA-seq analysis of iLM before and after distal and proximal co-culture.

a: RT-qPCR data showing fold change expression relative to day 0 iPSCs of additional MANC, ASM and mature smooth muscle markers in day 11 iLM cells before co-culture (grey), and in iLM after co-culture in distal (dark blue) and proximal (light blue) medium. N = 8. Bars show mean ± sd. P values were determined by paired, two-tailed Student's t

test. Significant ($p<0.05$) p values are highlighted in bold font. **b:** SPRING plot showing sample identity, Louvain clustering (resolution 0.1), cell cycle phase, expression of individual markers of interest, and expression of the LgM gene set in iLM before and after distal and proximal co-culture. **c:** Expression of genes found to be enriched in distal and proximal adult lung mesenchyme by Wang et al., 2018 in iLM before and after distal and proximal co-culture (250 genes each). Box plots indicate median (middle line), 25th and 75th percentile (box), 5th and 95th percentile (lines), as well as outlier. P values were determined by unpaired, two-tailed Student's t test. Significant ($p<0.05$) p values are highlighted in bold font. N = 1866 (pre co-culture iLM), 1236 (co-cultured distal), 1348 (co-cultured proximal).

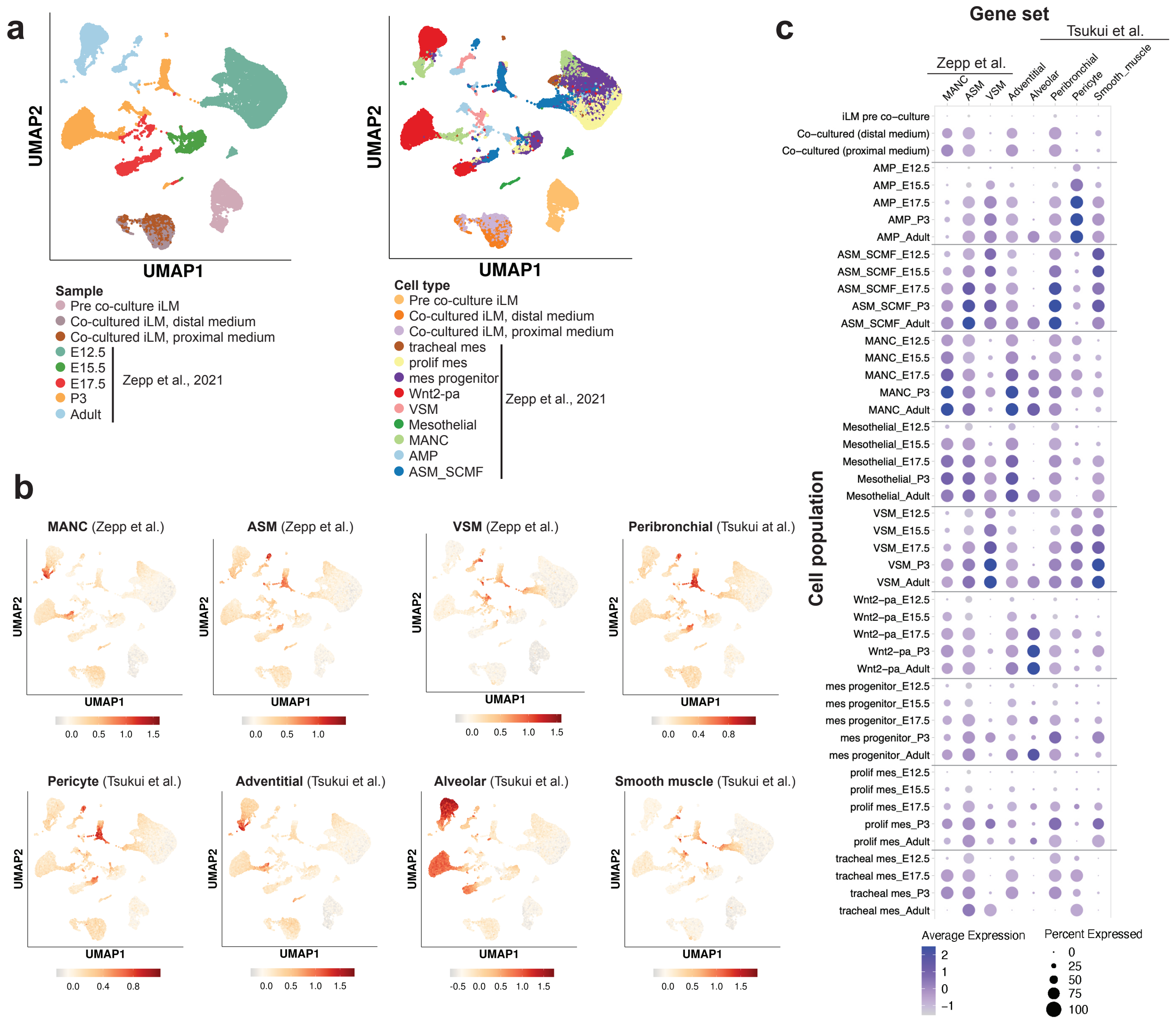

Supplementary Figure 8: scRNA-seq analysis of iLM before and after co-culture compared to published datasets of primary embryonic and adult mouse lung.
a: UMAP showing iLM before and after distal and proximal co-culture integrated (without harmonization) with a previously published lung mesenchyme scRNA-seq time series dataset by Zepp et al., 2021. Left plot shows sample identity, right plot shows cell type annotations by Zepp et al. NB: integration of new datasets with prior published datasets may be susceptible to technical batch effects. **b:** UMAPs showing expression of gene sets of interest by Zepp et al.,2021 (MANC, VSM, ASM) and Tsukui et al., 2020 (Adventitial, Alveolar, Peribronchial, Pericyte) in combined scRNA-seq datasets. **c:** Dot plots quantifying the expression of gene sets shown in Supplementary Fig. 8b.
