# Supplementary material for: Directed differentiation of mouse pluripotent stem cells into functional lung-specific mesenchyme
Source: Nat Commun. 2023 Jun 13;14:3488. doi: 10.1038/s41467-023-39099-9 (PMC10264380; doi:10.1038/s41467-023-39099-9)
Supplement: Supplementary file 3 — Description of Additional Supplementary Files [file 41467_2023_39099_MOESM3_ESM.pdf]

Description of Additional Supplementary Files

File Name: Supplementary Data 1
Description: **Differentially expressed genes in primary and iPSC-derived lung mesenchyme.** All differentially expressed genes in clusters 1-5.

File Name: Supplementary Data 2
Description: **Embryonic lung mesenchymal gene sets.** Gene set lists used to test for enrichment of lung mesenchymal genes. LgM gene set consists of genes previously suggested to be expressed in early lung mesenchyme, Han et al. gene set consists of a list of genes found to be enriched in E9.5 mouse lung mesenchyme by Han et al, 2020.

File Name: Supplementary Data 3
Description: **Top 50 enriched genes in MANC, ASM, and VSM cells in Zepp et al 2021.** Top genes enriched genes used for gene sets in Fig. 6.

File Name: Supplementary Data 4
Description: **Enriched genes in Acta2+ clusters. Top** 30 genes enriched in MANC, ASM and VSM cells in our scRNA-seq dataset.

File Name: Supplementary Data 5
Description: **Enriched genes in adult mouse distal and proximal lung from Wang et al., 2018.** Distal and proximal gene sets used in Fig. 6.

File Name: Supplementary Data 6
Description: **Top50 enriched genes in adult mouse lung cell types based on Tsukui et al., 2020.** Top genes enriched used for gene sets in Fig. 6.

File Name: Supplementary Data 7
Description: **Catalog numbers of probes used for RT-qPCR.**
