# Supplementary material for: Directed differentiation of mouse pluripotent stem cells into functional lung-specific mesenchyme
Source: Nat Commun. 2023 Jun 13;14:3488. doi: 10.1038/s41467-023-39099-9 (PMC10264380; doi:10.1038/s41467-023-39099-9)
Supplement: Supplementary file 11 — Reporting Summary [file 41467_2023_39099_MOESM11_ESM.pdf]

nature portfolio

Corresponding author(s): Darrell Kotton
Last updated by author(s): May 22, 2023

Reporting Summary

Nature Portfolio wishes to improve the reproducibility of the work that we publish. This form provides structure for consistency and transparency in reporting. For further information on Nature Portfolio policies, see our Editorial Policies and the Editorial Policy Checklist.

Statistics

For all statistical analyses, confirm that the following items are present in the figure legend, table legend, main text, or Methods section.

n/a	Confirmed	
☐	☒	The exact sample size (*n*) for each experimental group/condition, given as a discrete number and unit of measurement
☐	☒	A statement on whether measurements were taken from distinct samples or whether the same sample was measured repeatedly
☐	☒	The statistical test(s) used AND whether they are one- or two-sided<br>*Only common tests should be described solely by name; describe more complex techniques in the Methods section.*
☒	☐	A description of all covariates tested
☒	☐	A description of any assumptions or corrections, such as tests of normality and adjustment for multiple comparisons
☐	☒	A full description of the statistical parameters including central tendency (e.g. means) or other basic estimates (e.g. regression coefficient) AND variation (e.g. standard deviation) or associated estimates of uncertainty (e.g. confidence intervals)
☐	☒	For null hypothesis testing, the test statistic (e.g. *F*, *t*, *r*) with confidence intervals, effect sizes, degrees of freedom and *P* value noted<br>*Give P values as exact values whenever suitable.*
☒	☐	For Bayesian analysis, information on the choice of priors and Markov chain Monte Carlo settings
☒	☐	For hierarchical and complex designs, identification of the appropriate level for tests and full reporting of outcomes
☒	☐	Estimates of effect sizes (e.g. Cohen's *d*, Pearson's *r*), indicating how they were calculated

Our web collection on statistics for biologists contains articles on many of the points above.

Software and code

Policy information about availability of computer code

Data collection	No software was used for data collection.
Data analysis	Methods section includes a description with references of the following software packages used for scRNA-seq analaysis: Seurat v4.1.0, SPRING, Cell Ranger Pipeline v3.0.2, Louvain v0.4.3, SCTransform v0.3.5, MAST v1.20.1. Flow cytometry data was analyzed with FlowJo v10.7.1

For manuscripts utilizing custom algorithms or software that are central to the research but not yet described in published literature, software must be made available to editors and reviewers. We strongly encourage code deposition in a community repository (e.g. GitHub). See the Nature Portfolio guidelines for submitting code & software for further information.

Data

Policy information about availability of data

All manuscripts must include a data availability statement. This statement should provide the following information, where applicable:

- Accession codes, unique identifiers, or web links for publicly available datasets
- A description of any restrictions on data availability
- For clinical datasets or third party data, please ensure that the statement adheres to our policy

The scRNA-seq data reported in this publication have been deposited in NCBI's Gene Expression Omnibus. The scRNA-seq dataset comparing engineered to primary embryonic lung mesenchyme is available under accession code GSE203245 (https://www.ncbi.nlm.nih.gov/geo/query/acc.cgi?acc=GSE203245). The scRNA-seq dataset focusing on mouse lung Acta2+ cells is available under accession code GSE203243 (https://www.ncbi.nlm.nih.gov/geo/query/acc.cgi?acc=GSE203243). The

scRNA-seq dataset focusing on co-cultured iLM is available under accession code GSE228313 (https://www.ncbi.nlm.nih.gov/geo/query/acc.cgi?acc=GSE228313). GRC38m mouse genome (https://ftp.ncbi.nlm.nih.gov/genomes/all/GCF/000/001/635/GCF_000001635.27_GRCm39/GCF_000001635.27_GRCm39_genomic.fna.gz) was used as reference genome for sequence mapping. Source data are provided with this paper.

Human research participants

Policy information about studies involving human research participants and Sex and Gender in Research.

Reporting on sex and gender	This study does not involve any human research participants.
Population characteristics	This study does not involve any human research participants.
Recruitment	This study does not involve any human research participants.
Ethics oversight	This study does not involve any human research participants.

Note that full information on the approval of the study protocol must also be provided in the manuscript.

Field-specific reporting

Please select the one below that is the best fit for your research. If you are not sure, read the appropriate sections before making your selection.

☒ Life sciences ☐ Behavioural & social sciences ☐ Ecological, evolutionary & environmental sciences

For a reference copy of the document with all sections, see nature.com/documents/nr-reporting-summary-flat.pdf

Life sciences study design

All studies must disclose on these points even when the disclosure is negative.

Sample size	Sample sizes are delineated in the text or figure legends for each result. Sample sizes were selected based on variance data from pilot experiments, and a power calculation was performed using a commercially available power calculation tool (https://www.stat.ubc.ca/~rollin/stats/ssize/n2.html).
Data exclusions	No data was excluded for this study.
Replication	Each data point shown in this study represents an independent biological replicate. At least 3 biological replicates were collected for each experiment to ensure reproducibility. All results shown in this study were reproducible.
Randomization	Randomization was not relevant to this study, since for the experiments shown here the starting material (i.e. cells) was the same for all conditions and subsequently underwent different experimental treatment.
Blinding	Investigators were not blinded for this study, since all collected data was quantifiable and did not require subjective judgment or interpretation. For example, scRNAseq comparisons are performed computationally without regard to sample naming assignments. Likewise, for RT-qPCR CT values are scored quantitatively regardless of sample identity.

Reporting for specific materials, systems and methods

We require information from authors about some types of materials, experimental systems and methods used in many studies. Here, indicate whether each material, system or method listed is relevant to your study. If you are not sure if a list item applies to your research, read the appropriate section before selecting a response.

Materials & experimental systems

n/a	Involved in the study	
☐	☒	Antibodies
☐	☒	Eukaryotic cell lines
☒	☐	Palaeontology and archaeology
☐	☒	Animals and other organisms
☒	☐	Clinical data
☒	☐	Dual use research of concern

Methods

n/a	Involved in the study	
☒	☐	ChIP-seq
☐	☒	Flow cytometry
☒	☐	MRI-based neuroimaging

Antibodies

Antibodies used

chicken anti-GFP, Aves labs GFP-1010, 1/500; goat anti-PDGFRA, R&D Systems AF1062, 1/200; rat anti-SOX2, ThermoFisher 14-9811-82, 1/250; rat anti-E-cadherin, Invitrogen 13-1900, 1/200; rabbit anti-SMA, Abcam ab5694, 1/500; rabbit anti-proSFTPC, Abcam ab211326, 1/250; rabbit anti-SOX9, Abcam ab185966, 1/500; rabbit anti-TTF1 (Nkx2-1), Abcam ab76013, 1/500; rabbit anti-p63, Cell Signaling 13109, 1/500; goat anti-RFP, MyBioSource, MBS448122, 1/500; APC rat anti-mouse FLK-1, BD Biosciences 560070, 1/50; Alexa Fluor 647 Rat anti mouse CD144, BD Biosciences 562242, 1/50; BV421 Rat Anti-Mouse CD326, BD Biosciences 563214, 1/500, Alexa Fluor® 488 AffiniPure Donkey Anti-Chicken IgY (IgG) (H+L), Jackson ImmunoResearch 703-545-155, 1/500; Alexa Fluor® 647 AffiniPure Donkey Anti-Rabbit IgG (H+L), Jackson ImmunoResearch 711-605-152, 1/500; Cy™3 AffiniPure Donkey Anti-Goat IgG (H+L), Jackson ImmunoResearch 705-165-147, 1/500; Cy™3 AffiniPure Donkey Anti-Rat IgG (H+L), Jackson ImmunoResearch 712-165-153, 1/500; APC Rat IgG2a k Isotype control, BD Biosciences 553932, 1/50; Alexa Fluor® 647 Rat IgG2a, κ Isotype Control, BD Biosciences 557690, 1/50.

Validation

chicken anti-GFP, Aves labs GFP-1010: Validated by manufacturer. Antibodies were analyzed by western blot analysis (1:5000 dilution) and immunohistochemistry (1:500 dilution) using transgenic mice expressing the GFP gene product. Western blots were performed using BlokHen® (Aves Labs) as the blocking reagent, and HRP-labeled goat anti-chicken antibodies (Aves Labs, Cat. #H-1004) as the detection reagent. Immunohistochemistry used tetramethyl rhodamine-labeled anti-chicken IgY.
goat anti-PDGFRA, R&D Systems AF1062: Validated by manufacturer. PDGFRa was detected in immersion fixed frozen sections of mouse embryo using Goat Anti-Mouse PDGF Ra Antigen Affinity-purified Polyclonal Antibody at 15 µg/mL overnight at 4 °C.
rat anti-SOX2, ThermoFisher 14-9811-82: Validated by manufacturer. This antibody has been tested for immunohistochemistry in FFPE human testes at less than or equal to 5 µg/mL. The antibody can be used with either high or low pH antigen retrieval. This antibody has been tested by immunocytochemistry on fixed and permeabilized iPS cells at less than or equal to 5 µg/mL. Citations e.g. Alexandrova S, Kalkan T, Humphreys P, Riddell A, Scognamiglio R, Trumpp A, Nichols J. Selection and dynamics of embryonic stem cell integration into early mouse embryos. Development. 2016 Jan 1;143(1):24-34. doi: 10.1242/dev.124602. Epub 2015 Nov 19. PMID: 26586221; PMCID: PMC4725202.
rat anti-E-cadherin, Invitrogen 13-1900: Validated by manufacturer. Citations e.g. Shukla VC, Duarte-Sanmiguel S, Panic A, Senthilvelan A, Moore J, Bobba C, Benner B, Carson WE 3rd, Ghadiali SN, Gallego-Perez D. Reciprocal Signaling between Myeloid Derived Suppressor and Tumor Cells Enhances Cellular Motility and is Mediated by Structural Cues in the Microenvironment. Adv Biosyst. 2020 Jun;4(6):e2000049. doi: 10.1002/adbi.202000049. Epub 2020 May 18. PMID: 32419350; PMCID: PMC7489303.
rabbit anti-SMA, Abcam ab5694: Validated by manufacturer. Tested for IHC-P in PACT-sRIMS-cleared virgin and lactating mammary glands, mouse intestine and pancreas tissue, mouse aortic regurgitation, and mouse tonsil tissue. Citations e.g. Lloyd-Lewis B, Davis FM, Harris OB, Hitchcock JR, Lourenco FC, Pasche M, Watson CJ. Imaging the mammary gland and mammary tumours in 3D: optical tissue clearing and immunofluorescence methods. Breast Cancer Res. 2016 Dec 13;18(1):127. doi: 10.1186/s13058-016-0754-9. PMID: 27964754; PMCID: PMC5155399.
rabbit anti-proSFTPC, Abcam ab211326: Validated by manufacturer. Tested for immunohistochemical analysis of 4% paraformaldehyde-fixed, 0.2% Triton X-100 permeabilized frozen mouse lung tissue. Citations e.g. Fang Y, Shao H, Wu Q, Wong NC, Tsong N, Sime PJ, Que J. Epithelial Wntless regulates postnatal alveologenesis. Development. 2022 Jan 1;149(1):dev199505. doi: 10.1242/dev.199505. Epub 2022 Jan 10. PMID: 34931663; PMCID: PMC8881739.
rabbit anti-SOX9, Abcam ab185966: Validated by manufacturer. Tested for IHC-P in mouse eye tissue, rat, mouse and human colon tissue and human breast carcinoma tissue. Citations e.g. Wang Y, Zhang M, Huan Z, Shao S, Zhang X, Kong D, Xu J. FSH directly regulates chondrocyte dedifferentiation and cartilage development. J Endocrinol. 2021 Feb;248(2):193-206. doi: 10.1530/JOE-20-0390. PMID: 33295881.
rabbit anti-TTF1 (Nkx2-1), Abcam ab76013: Validated by manufacturer. Tested for IHC-P in human lung carcinoma and thyroid carcinoma tissue, rat lung tissue, mouse lung tissue. Citations e.g. Zewdu R, Mehrabad EM, Ingram K, Fang P, Gillis KL, Camolotto SA, Orstad G, Jones A, Mendoza MC, Spike BT, Snyder EL. An NKX2-1/ERK/WNT feedback loop modulates gastric identity and response to targeted therapy in lung adenocarcinoma. Elife. 2021 Apr 6;10:e66788. doi: 10.7554/eLife.66788. PMID: 33821796; PMCID: PMC8102067.
rabbit anti-p63, Cell Signaling 13109: Validated by manufacturer. This antibody has been validated using SimpleChIP® Enzymatic Chromatin IP Kits. Citations e.g. Zhang C, Lee HJ, Shrivastava A, Wang R, McQuiston TJ, Challberg SS, Pollok BA, Wang T. Long-Term In Vitro Expansion of Epithelial Stem Cells Enabled by Pharmacological Inhibition of PAK1-ROCK-Myosin II and TGF-β Signaling. Cell Rep. 2018 Oct 16;25(3):598-610.e5. doi: 10.1016/j.celrep.2018.09.072. PMID: 30332641; PMCID: PMC6284236.
goat anti-RFP, MyBioSource, MBS448122: Validated by manufacturer. Tested for immunofluorescence in 293HEK cells transfected with RFP; cells were fixed with 4% of PFA.
APC rat anti-mouse FLK-1, BD Biosciences 560070: validated by manufacturer. Tested for flow cytometric analysis of FLK-1 expression in bEnd.3 cells. Citations e.g. Kataoka H, Takakura N, Nishikawa S, Tsuchida K, Kodama H, Kunisada T, Risau W, Kita T, Nishikawa SI. Expressions of PDGF receptor alpha, c-Kit and Flk1 genes clustering in mouse chromosome 5 define distinct subsets of nascent mesodermal cells. Dev Growth Differ. 1997 Dec;39(6):729-40. doi: 10.1046/j.1440-169x.1997.t01-5-00009.x. PMID: 9493833.
Alexa Fluor 647 Rat anti mouse CD144, BD Biosciences 562242: Validated by manufacturer. Tested for flow cytometric analysis in mouse bEnd.3 cells (ATCC# CRL-2299). Citations e.g. Breier G, Breviario F, Caveda L, Berthier R, Schnürch H, Gotsch U, Vestweber D, Risau W, Dejana E. Molecular cloning and expression of murine vascular endothelial-cadherin in early stage development of cardiovascular system. Blood. 1996 Jan 15;87(2):630-41. PMID: 8555485.
BV421 Rat Anti-Mouse CD326, BD Biosciences 563214: Validated by manufacturer. Tested for flow cytometry in mouse thymocytes and splenic T lymphocytes. Birebent B, Somasundaram R, Purev E, Li W, Mitchell E, Hoey D, Bloom E, Mastrangelo M, Maguire H, Harris DT, Staib L, Braumuller H, Leeser C, Kuttner N, Beger HG, Herlyn D. Anti-idiotypic antibody and recombinant antigen vaccines in colorectal cancer patients. Crit Rev Oncol Hematol. 2001 Jul-Aug;39(1-2):107-13. doi: 10.1016/s1040-8428(01)00125-1. PMID: 11418307.
Alexa Fluor® 488 AffiniPure Donkey Anti-Chicken IgY (IgG) (H+L), Jackson ImmunoResearch 703-545-155: Validated by manufacturer. Based on immunoelectrophoresis and/or ELISA, the antibody reacts with whole molecule chicken IgY. It also reacts with the light chains of other chicken immunoglobulins. No antibody was detected against non-immunoglobulin serum proteins. The antibody has been tested by ELISA and/or solid-phase adsorbed to ensure minimal cross-reaction with bovine, goat, guinea pig, syrian hamster, horse, human, mouse, rabbit, rat and sheep serum proteins, but it may cross-react with immunoglobulins from other species.
Alexa Fluor® 647 AffiniPure Donkey Anti-Rabbit IgG (H+L), Jackson ImmunoResearch 711-605-152: Validated by manufacturer. Based on immunoelectrophoresis and/or ELISA, the antibody reacts with whole molecule rabbit IgG. It also reacts with the light chains of other rabbit immunoglobulins. No antibody was detected against non-immunoglobulin serum proteins. The antibody has been tested

by ELISA and/or solid-phase adsorbed to ensure minimal cross-reaction with bovine, chicken, goat, guinea pig, syrian hamster, horse, human, mouse, rat and sheep serum proteins, but it may cross-react with immunoglobulins from other species.
Cy™3 AffiniPure Donkey Anti-Goat IgG (H+L), Jackson ImmunoResearch 705-165-147. Validated by manufacturer. Based on immunoelectrophoresis and/or ELISA, the antibody reacts with whole molecule goat IgG. It also reacts with the light chains of other goat immunoglobulins. No antibody was detected against non-immunoglobulin serum proteins. The antibody has been tested by ELISA and/or solid-phase adsorbed to ensure minimal cross-reaction with chicken, guinea pig, syrian hamster, horse, human, mouse, rabbit and rat serum proteins, but it may cross-react with immunoglobulins from other species.
Cy™3 AffiniPure Donkey Anti-Rat IgG (H+L), Jackson ImmunoResearch 712-165-153: Validated by manufacturer. Based on immunoelectrophoresis and/or ELISA, the antibody reacts with whole molecule rat IgG. It also reacts with the light chains of other rat immunoglobulins. No antibody was detected against non-immunoglobulin serum proteins. The antibody has been tested by ELISA and/or solid-phase adsorbed to ensure minimal cross-reaction with bovine, chicken, goat, guinea pig, syrian hamster, horse, human, mouse, rabbit and sheep serum proteins, but it may cross-react with immunoglobulins from other species.
APC Rat IgG2a k Isotype control, BD Biosciences 553932: Validated by manufacturer. The R35-95 hybridoma was generated by hybridization of Y3 myeloma cells with spleen cells from LOU rats immunized with mouse immunoglobulins. The R35-95 hybridoma produces rat IgG2a, κ immunoglobulin that has no measurable reactivity with mouse immunoglobulins. The R35-95 immunoglobulin was selected as an isotype control following screening for low background binding on a variety of mouse and human tissues
Alexa Fluor® 647 Rat IgG2a, κ Isotype Control, BD Biosciences 557690: Validated by manufacturer. This antibody is routinely tested by flow cytometric analysis. Other applications were tested at BD Biosciences Pharmingen during antibody development only or reported in the literature.

Eukaryotic cell lines

Policy information about cell lines and Sex and Gender in Research

Cell line source(s)	The Tbx4-LER reporter mouse iPSC line was generated by reprogramming tail tip fibroblasts from a female mouse as described in Figure 1 and Supplementary Figure 1. The Nkx2-1mCherry reporter ESC line is male and has been previously described (Bilodeau et al., Stem Cell Reports 2014 and Kurmann et al, Cell Stem Cell 2014) and was a generous gift from Dr. Janet Rossant (SickKids Research Institute, Toronto, Canada).
Authentication	For the Tbx4-LER line a normal karyotype was confirmed and pluripotency was tested by alkaline phosphatase stain. The Nkx2-1mCherry line is published and was found to be euploid by G-banding.
Mycoplasma contamination	All cell lines used were tested and were negative for mycoplasma contamination.
Commonly misidentified lines (See ICLAC register)	This study did not involve any commonly misidentified lines.

Animals and other research organisms

Policy information about studies involving animals; ARRIVE guidelines recommended for reporting animal research, and Sex and Gender in Research

Laboratory animals	C57BL/6J mouse line (Jackson Laboratory, #000664); Tbx4-LER mouse line Zhang et al. BMC Biology 2013. This line was generated in the lab of Wei Shi (University of Southern California); Acta2hrGFP/Cspg4-Cre-LSL-dsRed mouse line, Paez-Cortez et al, Plos ONE 2013. This line was a generous gift from the lab of Alan Fine (Boston University); Sftpc-CreERT2/Rosa-TdTomato mouse line, Rock et al., PNAS 2011. To generate the Sftpc-Tomato animals, Sftpc-CreERT2 (Jackson Laboratory, #028054) males were crossed with Rosa-tdTomato females (Jackson Laboratory, #007914); Pdgfra-GFP mouse line, Hamilton et al., Mol. Cell. Biol. 2003 (Jackson Laboratory, #007669); Nkx2-1GFP mouse line, Longmire et al., Cell Stem Cell 2012. This line was previously generated in our lab and is also commercially available (Jackson Laboratory, #066764-JAX). Mice were maintained in controlled environment at 18-25°C and 30-70 % humidity with a light/dark cycle of 12/12 hours. 2-6 months old animals were used for timed pregnancies, 3-6 months old animals were used for isolation of adult mouse lung tissue.
Wild animals	The study did not involve wild animals.
Reporting on sex	Sex-based analysis was not performed in this study, since most experiments involved isolation of embryonic tissue, where determination of the sex of the embryos was not possible.
Field-collected samples	Study did not involve field-collected samples.
Ethics oversight	All experiments involving mice were approved by the Institutional Animal Care and Use Committee of Boston University School of Medicine.

Note that full information on the approval of the study protocol must also be provided in the manuscript.

Flow Cytometry

Plots

Confirm that:

☒ The axis labels state the marker and fluorochrome used (e.g. CD4-FITC).

☒ The axis scales are clearly visible. Include numbers along axes only for bottom left plot of group (a 'group' is an analysis of identical markers).

☒ All plots are contour plots with outliers or pseudocolor plots.

☒ A numerical value for number of cells or percentage (with statistics) is provided.

Methodology

Sample preparation	Cultured adherent cells were dissociated using 0.05% Trypsin for 4 min and subsequently neutralized using Fetal Bovine Serum. Epithelial-mesenchymal co-cultures were incubated in 2 mg/ml Dispase to dissociate the 3D Matrigel and subsequently incubated in 0.05 % Trypsin for 10 min, followed by addition of Fetal Bovine Serum. Primary embryonic tissue was dissociated using TrypLE (Gibco 12640-013). If an antibody stain was used, cells were incubated in FACS buffer (PBS with 2 % FBS) containing antibody for 30 min on ice, washed once with FACS buffer and resuspended in FACS buffer for analysis.
Instrument	Cells were analyzed using a Stratedigm S1000EXi instrument. For sorting, cell were sorted on a Beckman Coulter MoFlo Astrios instrument.
Software	Flow Cytometry data was analyzed using the FlowJo software.
Cell population abundance	In general, 50'000 to 100'000 cells were collected for further analysis (e.g. RT-qPCR). For some experimental conditions with poor outgrowth, lower cell numbers (10'000 to 20'000 cells) were collected. Purity was determined by Flow Cytometry analysis after sorting for 1-2 replicates of each experiment. Purity rates were > 97% for all samples tested.
Gating strategy	All samples were gated for cells using FSC/SSC, single cells using FSC H/FSC A, and live cells using either Calcein blue or DRAQ7. Positive and negative staining populations were determined using an isotype control where an antibody stain was used. For fluorescently labeled proteins, positive and negative populations were determined using cells that did not express the fluorescent protein of interest (e.g. non-dox induced cells for the Tbx4-LER reporter, see Figure 1d). An example for the gating strategy is shown in Supplementary Figure 1f.

☒ Tick this box to confirm that a figure exemplifying the gating strategy is provided in the Supplementary Information.
